# Estimating the willingness-to-pay to avoid the consequences of foodborne illnesses: a discrete choice experiment

**DOI:** 10.1007/s10198-022-01512-3

**Published:** 2022-09-08

**Authors:** Kathleen Manipis, Brendan Mulhern, Philip Haywood, Rosalie Viney, Stephen Goodall

**Affiliations:** Centre for Health Economics Research and Evaluation, University of Technology Sydney, PO Box 123, Broadway, Sydney, NSW 2007 Australia

**Keywords:** Discrete choice experiment, Productivity, Foodborne illness, Willingness-to-pay, Compensation, Sick leave, I12

## Abstract

Lost productivity is one of the largest costs associated with foodborne illness (FBI); however, the methods used to estimate lost productivity are often criticised for overestimating the actual burden of illness. A discrete choice experiment (DCE) was undertaken to elicit preferences to avoid six possible FBIs and estimate whether ability to work, availability of paid sick leave and health-related quality of life affect willingness-to-pay (WTP) to avoid FBI. Respondents (*N* = 1918) each completed 20 DCE tasks covering two different FBIs [gastrointestinal illness, flu-like illness, irritable bowel syndrome (IBS), Guillain–Barre syndrome (GBS), reactive arthritis (ReA), or haemolytic uraemic syndrome (HUS)]. Attributes included: ability to work, availability of sick leave, treatment costs and illness duration. Choices were modelled using mixed logit regression and WTP was estimated. The WTP to avoid a severe illness was higher than a mild illness. For chronic conditions, the marginal WTP to avoid a chronic illness for one year, ranged from $531 for mild ReA ($1412 for severe ReA) to $1025 for mild HUS ($2195 for severe HUS). There was a substantial increase in the marginal WTP to avoid all the chronic conditions when the ability to work was reduced and paid sick leave was not available, ranging from $6289 for mild IBS to $11,352 for severe ReA. Including factors that reflect productivity and compensation to workers influenced the WTP to avoid a range of FBIs for both acute and chronic conditions. These results have implications for estimating the burden and cost of FBI.

## Introduction

Foodborne illnesses (FBIs) are very common, with an estimated two billion cases per year worldwide [1]. Foodborne illness can result in gastrointestinal (GI) and non-gastrointestinal illnesses, and can have serious long-term chronic sequelae [2, 3]. While mild cases are typically of short duration, self-resolving or managed with over-the-counter medications [1, 4], sequelae of serious cases of FBI may require intensive treatment over a long period of time [1, 2, 4].

Most FBIs occur unexpectedly, and the focus of intervention is on prevention through food safety and on management of side-effects. Accurately estimating the burden and cost of FBI is important for the development and prioritising of effective food safety policy and treatments. In Australia and the UK, the annual cost of FBIs has been estimated at AUD$1.2 billion (USD $860 million) and £1.9 billion (USD $2.4 billion) respectively [5, 6]. These estimates included costs, such as health care use and lost productivity. Productivity losses are one of the main drivers of cost, where double counting or omission of health impacts remains a key issue [5, 7–9]. Productivity is the output per unit of input of capital or labour and measures the contribution of individual workers to a firm [10]. If a worker is unfit to work there is a loss of productivity (reduced output), and under the assumptions of a competitive market, this can be measured by the wage of the worker which reflects the value of the marginal product [12, 31–33]. There may also be a loss of wages to the individual who is unable to work due to illness, but this may not capture the full cost to the individual because it does not capture the disutility of being ill.

The willingness-to-pay (WTP) to avoid a FBI is another measure that could be used within a cost–benefit analysis framework and help inform decision making. The individual’s WTP should in theory capture the value to them of the lost productivity (in the form of foregone wages), as well as the disutility of illness and any out of pocket costs of treatment. However, established labour market structures and compensation mechanisms such as paid sick leave entitlements in Australia, may affect WTP estimates [11]. Having paid sick leave entitlements means time can be taken off work without financial loss to the individual, but these costs are generally borne by the employer [8, 12]. Therefore it is important to consider the effect of paid sick leave in any WTP estimates.

One method available to assess population level preferences and measure WTP is through a discrete choice experiment (DCE) [13]. DCEs are a stated choice method where alternative scenarios are described in terms of various features (attributes) and respondents are asked to choose their preferred option from those presented. These choices can then modelled to estimate preferences for each level of each attribute presented in the experiment. DCEs have become widely used in health economics [14, 15]. By including a cost attribute the WTP for a good or service can be estimated. In previous research, DCE methods have been used to assess respondent preferences for risk of contracting campylobacter via food or water [16], and to assess the negative impacts of irritable bowel syndrome (IBS) (though without the specific context of FBIs) [17].

In this study we develop and implement a DCE with the aim of estimating WTP to avoid a range of acute and chronic FBIs, to inform cost estimates of different FBIs. The WTP to avoid an illness provides a measure of the opportunity cost associated with the illness because it captures both the impacts on QoL and the opportunity cost of time. A further aim of this study is to estimate whether ability to work, availability of paid sick leave and quality of life (QoL) affect the WTP to avoid different FBIs. To our knowledge this is the first DCE to examine preferences to avoid the negative aspects of both acute FBIs and the chronic sequelae.

A variety of pathogens cause FBI, including *Campylobacter* spp., Shiga toxin-producing *E. coli*, *Salmonella enterica*, *Salmonella enterica* ser. Typhi, *Shigella* spp., *Yersinia entercolitica*, norovirus, *Listeria monocytogenes*, and *Toxoplasma gondii* [18]. Common symptoms are nausea, vomiting, and diarrhoea. Symptoms may differ among the pathogens and some FBI may become severe and life-threatening. In this experiment, six common conditions were chosen, two acute illnesses: GI illness and flu-like illness; and four chronic illnesses: IBS, Guillain–Barre syndrome (GBS), reactive arthritis (ReA), and haemolytic uraemic syndrome (HUS). These chronic illnesses were considered to be the prominent sequelae of foodborne infection [2, 3] with long-term health impacts, and have been considered in several burden of FBI studies [4, 5, 9, 19, 20].

## Methods

We designed a DCE that described typical FBIs and their consequences as attributes. The theoretical framework for DCE techniques is based on Lancaster’s economic theory of consumer choice and McFadden’s random utility theory framework [21, 22]. The survey instrument was developed using the steps recommended by the International Society for Pharmacoeconomics and Outcomes Research (ISPOR) taskforce [23].

### Survey development

The DCE tasks were developed, assessed and revised in stages using qualitative and quantitative methods. Existing literature and clinical input were used to develop the vignettes, attribute descriptions and levels. Several studies have investigated the burden of FBIs in developed countries, including a number of studies that have considered QoL (or utility) effects [4, 5, 9, 19, 20]. The utility studies [24–27] used to inform burden of illness studies were reviewed to understand the characteristics that underlie health impacts of FBIs, and assist in developing vignettes and attribute descriptions.

The vignettes, attribute descriptions and levels were assessed and refined based on detailed feedback from a focus group, comprising researchers with experience in implementation of DCEs, and review by a medical expert to ensure descriptions were comprehensive and accurate. Before implementation of the DCE, the funder (Food Standards Australia and New Zealand), also arranged for an external peer review. The DCE survey was pilot tested in a general population sample of Australians (*N* = 200), focussing on the functionality of the survey system, the randomisation procedure, and clarity of the instructions (assessed using feedback questions). The survey contained three sections: (1) demographic information to ensure representativeness in terms of age group, gender, and location by state and territory; (2) the DCE task which comprised a total of 20 choice sets per respondent, with respondents randomised to two different illnesses; and (3) supplementary demographic questions about employment, health, and debriefing questions. Within the choice task section respondents were presented with 10 DCE tasks for the first illness and 10 DCE tasks for the second illness. For each illness, two health states of differing severity were presented (five mild and five severe). A summary of the survey flow is provided in the Appendix (Fig. 4).

### DCE task development

**Fig. 1:**
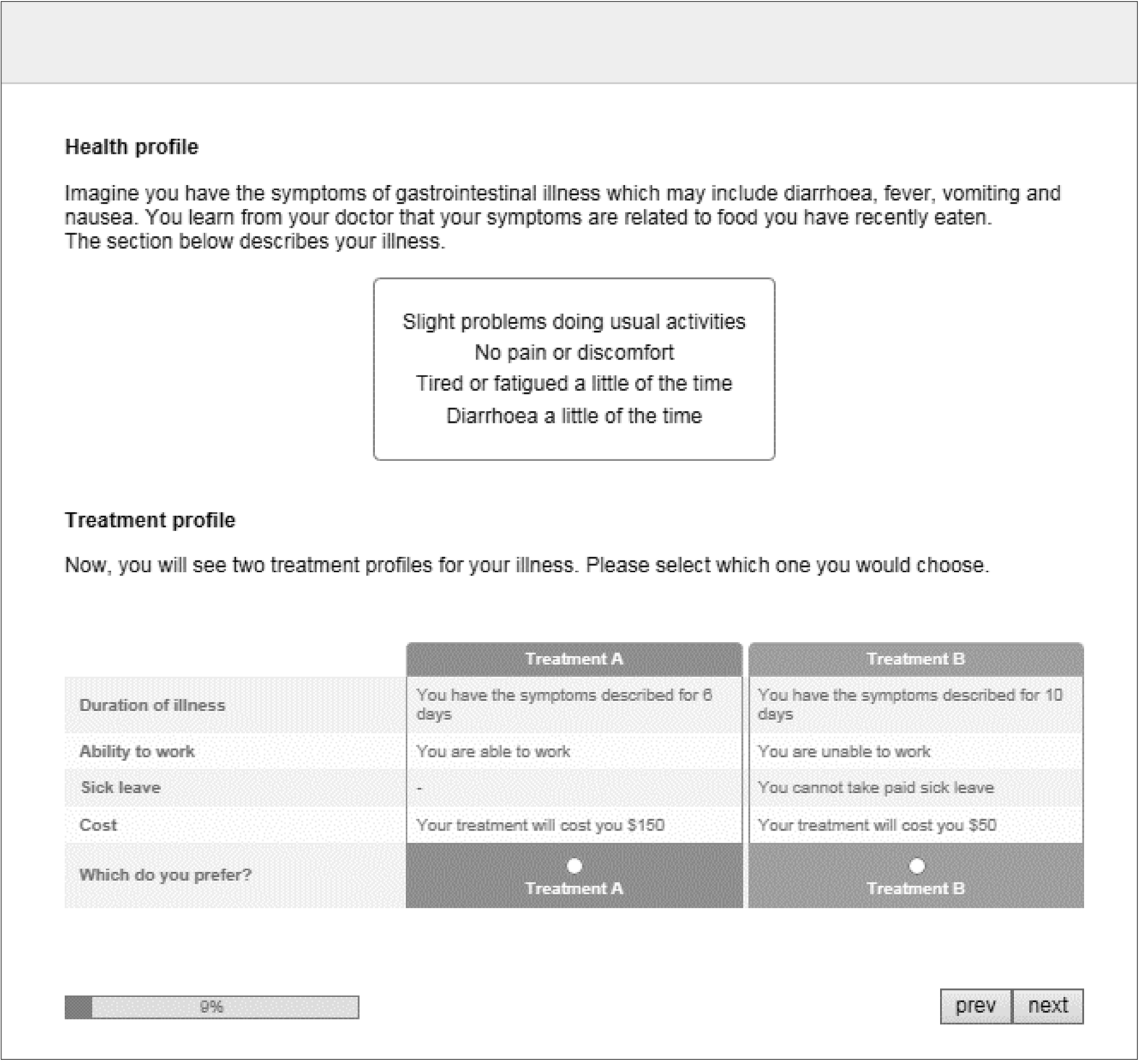
Example discrete choice experiment

Respondents were presented with a series of choice tasks describing a specific FBI profile and were asked to select the most preferred of two treatment options. An example of a DCE task is presented in Fig. 1. Each choice task incorporated three components: (1) a vignette describing the illness, (2) a health state profile describing the QoL and illness severity, and (3) a choice set describing two treatments and work profiles, defined by duration of illness, cost of treatment, and paid sick leave availability/ability to work (Table 1). Respondents were asked to imagine having the illness and health state as described in the vignettes, compare the attributes in the choice set, and then select the most preferred treatment to help the respondent return to normal health.

**Table 1:** Attributes and levels

Attribute	Description	Levels	A priori expectations
Acute illness			
Duration of illness	The duration of symptoms for an illness	You have the symptoms described for 1 day	A negative preference for a longer duration was expected
	Treatment A was always restricted to be of shorter duration than Treatment B	You have the symptoms described for 3 days	
		You have the symptoms described for 6 days	
		You have the symptoms described for 8 days	
		You have the symptoms described for 10 days	
Costs	The out of pocket costs due to the illness	Your treatment will cost you $0	A negative preference for a higher cost was expected
	Treatment A was always restricted to be more expensive than Treatment B	Your treatment will cost you $50	
		Your treatment will cost you $100	
		Your treatment will cost you $150	
		Your treatment will cost you $200	
		Your treatment will cost you $250	
Sick leave^a^	Time off work an employee can use for a health issue without losing pay	You are able to work	No a priori information imputed
	No restrictions were applied for this parameter	You are unable to work and can take paid sick leave	A positive preference for being able to work, followed by being able to take paid sick leave is expected
		You are unable to work and cannot take paid sick leave	
Chronic illness			
Duration of illness	The duration of symptoms for an illness	You have the symptoms described for 1 year	A negative preference for a longer duration was expected
	Treatment A was always restricted to be of shorter duration than Treatment B	You have the symptoms described for 3 years	
		You have the symptoms described for 6 years	
		You have the symptoms described for 8 years	
		You have the symptoms described for 10 years	
Costs	The out of pocket costs due to the illness	Your treatment will cost you $0	A negative preference for a higher cost was expected
	Treatment A was always restricted to be more expensive than Treatment B	Your treatment will cost you $2000	
		Your treatment will cost you $5000	
		Your treatment will cost you $10,000	
		Your treatment will cost you $15,000	
Sick leave^a^	Time off work an employee can use for a health issue without losing pay	You are able to work	The first and last levels are ordered correctly, however it is unclear how the other levels should be ordered. There are trade-offs between the levels
	No restrictions were applied for this parameter	You are unable to work some of the time and can take paid sick leave	
		You are unable to work some of the time and cannot take paid sick leave	
		You are unable to work most of the time and can take paid sick leave	
		You are unable to work most of the time and cannot take paid sick leave	

^a^In the DCE, the sick leave attribute was split into two descriptors, ‘ability to work’ and ‘sick leave’

#### Vignette

The vignette consisted of background information on one of the six FBIs (Appendix Table 4) and a specific health state profile incorporating QoL and illness severity dimensions (mild or severe) (Appendix Fig. 5).

As part of the background information, a brief description of the symptoms was provided for the two acute illnesses on the basis that these are common with well understood symptoms for the general population. More detailed descriptions were used for the chronic illnesses because these are less familiar to the general population. Descriptions of the illnesses were reviewed by the medical expert and focus groups.

#### Health state profile

Descriptors of QoL and illness severity were used to differentiate the two health state profiles of the mild and severe cases of each illness. Each health state profile incorporated QoL and illness severity dimensions. The descriptions of the health state profiles for each illness are provided in the Appendix. The descriptions used in the survey to convey the health states to participants was based on validated questionnaires (EQ-5D [28, 29], SF-36 and SF-6D [30, 31], IBS-QoL [32]), which use phrases and statements that have previously been tested with the general population. Severity levels for each condition were reviewed by a medical expert to ensure the language used to describe the health state was consistent with the signs and symptoms of each condition and comprehensible for a layperson.

#### Attributes

To estimate WTP a monetary attribute needs to be included in the choice experiment. In this experiment this was achieved by including an attribute which described the costs of treatments which could reduce the duration of the episode of illness.

The levels for durations of illness were informed by the literature [2–4] and input from a medical expert. The duration of illness ranged from 1 to 10 days for an acute illness, and from 1 to 10 years for a chronic illness. The levels for the cost attribute were separately defined based on the duration and severity of the illness being valued and were intended to be realistic to respondents. A five-level attribute described the cost of treatment of the acute conditions (range $0 to $250), and a four-level attribute described the cost of treatment of the chronic conditions (range $0 to $15,000). Costs were presented to respondents as the amount that they would pay for treatment.

The ability to work and sick leave attributes were assigned three levels for the acute conditions and five levels for the chronic condition. This was to account for the different characteristics over a longer time horizon. The description of time spent being unable to work was based on the social functioning domain of the SF-36 and SF-6D [30, 31]. Combinations of the ability to work were coupled with availability of paid and unpaid sick leave to explore how respondents consider illness and loss of income simultaneously. The ability to work and sick leave attribute were presented as two separate descriptions in the choice sets to help respondents compare between the components.

#### Designed experiment

Two designs containing 30 choice sets were selected [33], one for the acute conditions and one for the chronic conditions. D-optimal methods was used to select choice sets using the design software NGene [34] to allow for estimation of the main effects (duration of illness, cost of treatment, and ability to work/sick leave) of a multinomial logit (MNL) model. Duration of illness and cost of treatment were fitted as continuous variables, and ability to work/sick leave was coded as a categorical variable in the design. The choice sets were constructed to force respondents to trade-off between duration of illness and cost, such that the profile for Treatment A in the choice set was always more expensive and had a shorter duration than Treatment B. This ensured that choice sets were not dominated. No restrictions were placed on the sick leave/ability to work attributes. The same design was used across the mild and severe health state profiles to explore how respondents trade based on the severity of the health state.

The sample size was calculated based on the rule-of-thumb to determine the minimum sample size by Johnson and Orme (1996); *N* > 500*c/(t*a), where, *c* = the largest number of levels of any one attribute; *t* = no. of tasks; *a* = no. of alternatives per task. For this DCE, the minimum sample size for one mild or severe illness was *N* > [500*6/(5*2)] = 300. Each illness was to be completed by approximately 600 respondents (total *n* = 1800), to provide 6000 observations per illness. This is consistent with the number of observations suggested for valid estimates of DCE parameters [35].

### Data collection

Two thousand respondents were recruited (200 for the pilot and 1800 for the main data collection) from an online panel (Toluna Australia). Invitations to participate were sent via email, and respondents were recruited consecutively, then randomised to two different conditions. A small monetary incentive was provided to respondents completing the survey in more than the preset minimum completion time of three minutes. Quotas for age, sex and location by state and territory, were established to ensure comparability to the Australian population.

### Data analysis

Data were analysed using Stata software version 15.1. Descriptive statistics were used to analyse the characteristics of the overall sample. Two approaches were used to account for heterogeneity; mixed logit (MXL) and latent class (LC) modelling.

Mixed logit modelling relaxes the constraint that the coefficients are the same for all individuals and allows for flexible substitution between alternatives [36]. Two models for each illness (mild and severe) were estimated using the ‘mixlogit’ command in Stata, which takes into account repeated observations per respondent [36]. Duration of illness and ability to work/sick leave were specified as random variables in the models. Cost and duration of illness variables were coded as continuous variables, and the ability to work/sick leave was coded as dummy variables. Standard errors were clustered by respondent. Each model was simulated with 500 Halton draws. The reference level for each attribute was used to compare the estimated coefficients (1 day or 1 year; $0; and ‘You are able to work’).

Latent class modelling is used to classify responses into a distinct number of classes [37], where preferences are similar within a class but differ across the classes. Latent class models for each illness were estimated using the ‘lclogit’ command in Stata [37]. The number of classes to include was informed by the model with the lowest BIC [38] across models ranging from two to eight classes. Characteristics that are likely to affect preferences and WTP are employment status and income. Given these characteristics affecting WTP, the variables used inform class membership were age (less than 45 years; 45 years and older), gender (male; female), income (less than $52,000 per annum; $52,000 per annum or more), employment status (employed; not employed) and sick leave (no sick leave; paid sick leave).

The marginal WTP for each part-worth utility was derived using coefficients from each mixed logit model using the cost of treatment attribute to calculate a marginal rate of substitution (MRS), that is, the change in the cost attribute that would compensate for a change in another attribute [39]. The WTP to avoid a health state for each illness is relative to the respondents’ perception of ‘no foodborne illness’ or ‘full health’. The differences in the value between the severe and mild health states provides information about certain characteristics of the state descriptions used for the illnesses and provides an indication of the perceived severity order of the conditions relative to the health states described. To estimate the WTP to avoid an illness, the marginal WTP for the duration and ability to work/sick leave level are added for each health state. For example, the WTP to avoid one day of a severe case of GI illness when sick leave is not paid would be $176 (i.e., $32 + $144).

## Results

### Population

The survey was completed by 2022 respondents (response rate 45.8%). Analyses were conducted on respondents who completed the full survey (*N* = 1918). The majority (76%) of the sample agreed or strongly agreed that they considered the whole description whilst completing the tasks.

The demographic characteristics of the sample are summarised in Table 2 and Table 3. Respondent demographics characteristics were similar across all conditions. Compared to the national average, respondents were slightly less likely to be born overseas and had a higher level of educational attainment. Over half of the respondents were employed (54%), and of those 62% reported working full time in the past week; 77% reported having paid sick leave entitlements. Respondents in our sample on average had lower incomes (AUD $600 to $799 per week) than the Australian median (AUD $1012 per week) [40]. Approximately two fifths of the sample had prior experience of an acute FBI.

**Table 2:** Demographics: comparison of respondents and the Australian population

	Completers (*N* = 1918) *n* (%)	Australian population^a^
Age, years		
18–24	194 (10)	13.2%^b^
25–34	338 (18)	14.8%
35–44	351 (18)	13.4%
45–54	363 (19)	13.1%
55–64	311 (16)	11.5%
65 and over	361 (19)	15.2%
Gender, male, *n* (%)	876 (46)	49.7%
Residential state in Australia		
Australian Capital Territory	30 (2)	1.6%
New South Wales	589 (31)	32.0%
Northern Territory	16 (1)	1.0%
Queensland	401 (21)	20.1%
South Australia	162 (8)	7.1%
Tasmania	48 (3)	2.2%
Victoria	491 (26)	25.2%
Western Australia	181 (9)	10.8%
Country of birth, Australia	1465 (76)	71.5%
Highest level of education completed		
Primary or secondary	597 (32)	43.0%
Trade certificate/diploma	600 (31)	20.8%
Bachelor’s degree	528 (28)	17.0%
Higher degree	193 (10)	5.5%

^a^Source: Australian Bureau of Statistics [41, 42]

^b^Includes age groups 15–19 year old and 20–24 year old

**Table 3:** Income and employment status

	Completers (*N* = 1918) *n* (%)
Employed	1041 (54)
Self-employed	172 (9)
Working for an employer	869 (45)
Entitled or eligible to receive sick leave	672 (77)
Gross income, per year	
$52,000 or more	633 (33)
$20,800—$51,999	614 (32)
$1—$20,799	356 (19)
Other, nil income, negative income, prefer not to say	315 (16)

### Preference weights

#### Mixed logit models

**Fig. 2:**
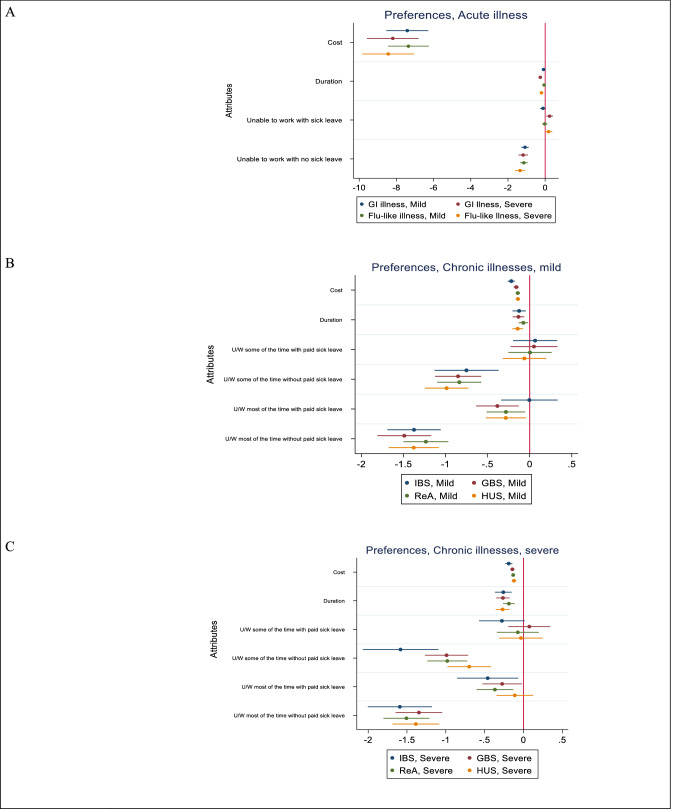
Preference estimations (95% CI). *CI* confidence interval, *GBS* Guillain Barre syndrome, *GI* gastrointestinal, *HUS* haemolytic uraemic syndrome, *IBS* irritable bowel syndrome, *PSL* paid sick leave, *ReA* reactive arthritis *U/W* unable to work

The results for each MXL model are presented for each illness in Fig. 2 and in the Appendix (Table 5, 6). For all illnesses, respondents preferred lower costs for treatment and shorter durations of illness. The coefficients for treatment costs and duration of illness were significant for all illnesses (*p* < 0.01). The availability of paid sick leave affected preferences. Respondents would prefer unable to work with a case of severe acute GI if paid sick leave were available (*p* = 0.009), compared with being able to work. The standard deviations for all random coefficients in the acute illness models (except for flu-like illness, unable to work with paid sick leave), indicated that there is heterogeneity in the preferences across the sample.

For the chronic illnesses, the coefficients for being unable to work some of the time when paid sick leave was available were not significant. There was significant heterogeneity in preferences when paid sick leave were available and faced with being unable to work some of the time for mild GBS and severe HUS and being unable to work most of the time for severe IBS, severe GBS and mild HUS.

The results indicate that respondents had the strongest preference to avoid being ill and unable to work when sick leave was not available. In all models, coefficients for being ill without paid sick leave were statistically significant (*p* < 0.01). Except for severe GBS, severe ReA, and mild HUS, the standard deviations were statistically significant for the coefficients where sick leave was not paid, indicating a variation in the preference estimates with respect to being unable to work when sick leave pay is not available.

The overall results for the ability to work and sick leave attribute in the chronic illness health states were ordered as expected. Being able to work was preferred to being unable to work, and being unable to work some of the time was preferred to being unable to work most of the time. This pattern was consistent across all models.

#### Latent class models

The results for each LC model are presented for each illness in the Appendix (Tables 7, 8, 9). Based on the BIC estimates the best fit was the model with three classes for all illnesses, other than for mild and severe flu-like illness (the best fit was four classes), and mild GBS (two classes). As the improvements in BIC estimates for these models were minimal (less than 1%) compared with the three class models, the models with three classes are presented for all illnesses for consistency. Across the 12 illnesses no clear pattern was discernible, although, there were differences in preferences for duration of illness and costs observed.

The LC model for the mild flu-like illness was able to clearly distinguish between differences in the underlying taste pattern (mean posterior probability, 0.820). Class 1 (share, 47.9%) were more likely to be male (*p* < 0.05) and older (*p* < 0.05) compared with class 3 (share, 17.8%). Class 1 prefers being well to not being ill for any duration (*p* < 0.001), not paying for treatment costs (*p* < 0.001), and prefers not being unable to work without paid sick leave (*p* < 0.001). Class 2 (share, 34.2%) were more likely to be older (*p* < 0.05) and not employed (*p* < 0.05) compared with class 3. Class 2 strongly prefers not paying for treatment costs (*p* < 0.001); surprisingly, class 2 prefers being ill to being well (*p* < 0.001). Preferences estimates for the ability to work and the availability of sick leave attribute were not statistically significant for class 2.

In the LC model for ReA, Class 1 (share, 35.3%) were more likely to be older (*p* < 0.05) than class 3 (share, 51.3%). Class 1 preferred being ill to being well for any duration (*p* < 0.05) and preferred not paying for treatment costs (*p* < 0.01). Class 1 preferred being unable to work some of the time with paid sick leave, however strongly preferred not being unable to work most of the time without paid sick leave. Class 2 (share, 13.4%) were more likely to be younger in age compare with class 3, and strongly preferred not being ill for any duration (*p* < 0.001). In the reference, class 3, preferences were ordered such that respondents preferred not being ill for any duration (*p* < 0.001), not paying for treatment costs (*p* < 0.001), and they preferred being able to work compared with not being able to work some or most of the time, with or without sick leave.

### Willingness-to-pay to avoid an illness

**Fig. 3:**
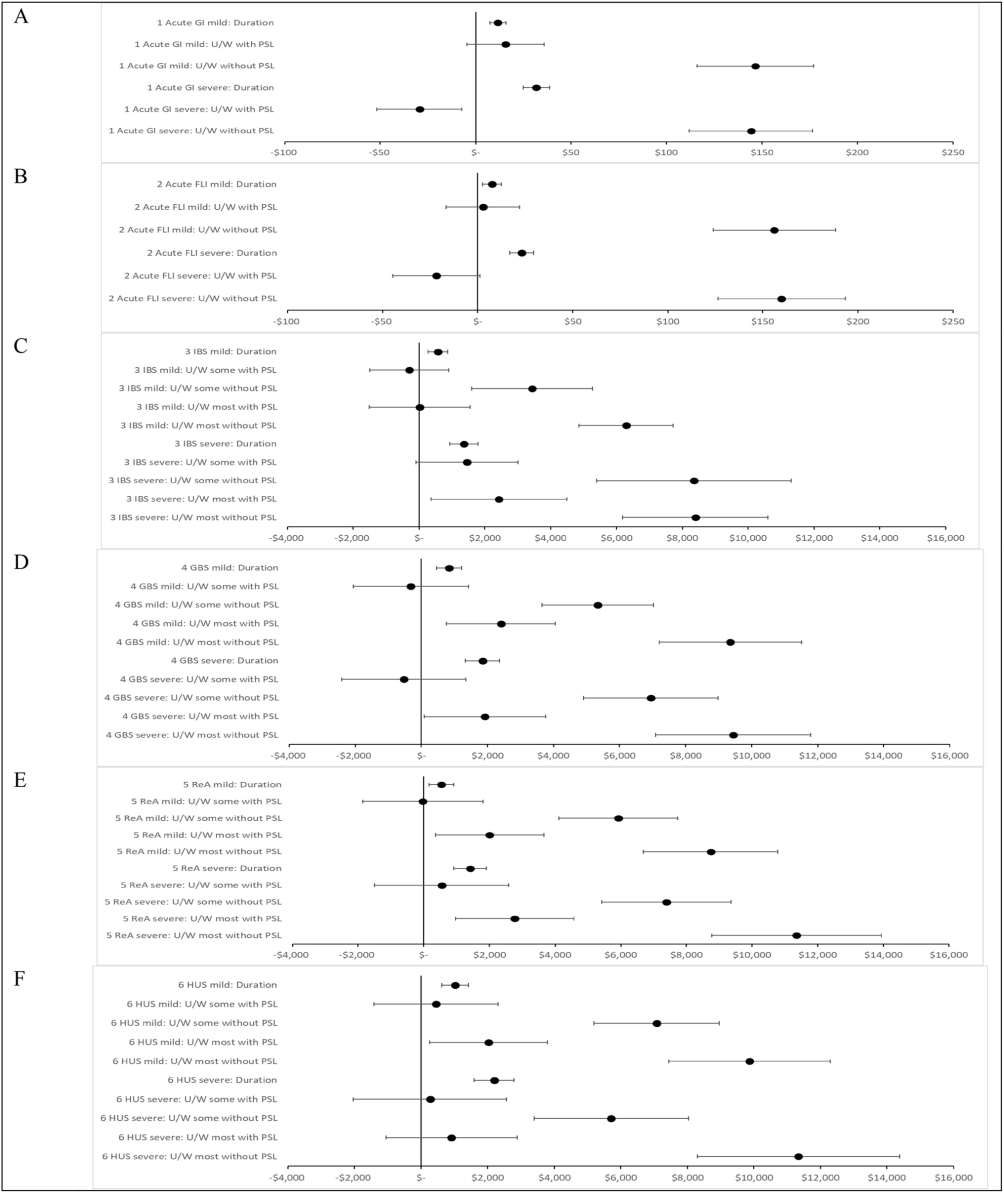
mWTP (95% CI) ($AUD 2017). *CI* confidence interval, *FLI* flu-like illness, *GBS* Guillain Barre syndrome, *GI* gastrointestinal, *HUS* haemolytic uraemic syndrome, *IBS* irritable bowel syndrome, *mWTP* marginal willingness-to-pay, *PSL* paid sick leave, *ReA* reactive arthritis, *U/W* unable to work

**Fig. 4:**
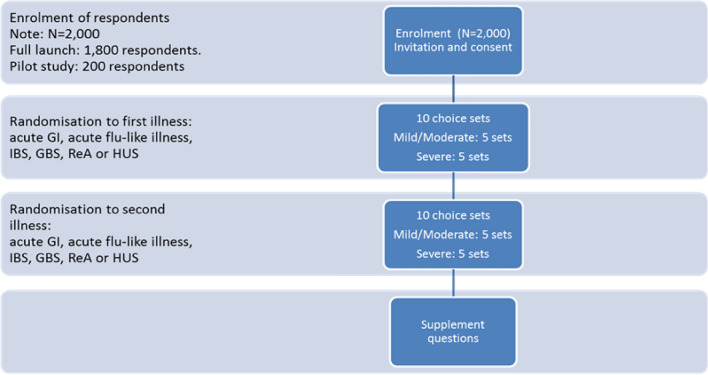
Survey layout

The marginal WTP values for each attribute are presented in Fig. 3, and Appendix Tables 7, 10, 11. Results are reported in Australian dollars in 2017. The marginal WTP to avoid a mild acute GI illness for one day was $12 and $32 for a severe acute GI illness. The results for flu-like illness were similar. The marginal WTP to avoid a mild chronic illness for one year ranged from $531 for ReA to $1025 for HUS, and for a severe chronic illness ranged from $1367 for IBS to $2195 for HUS.

The increase in the WTP is generally larger when transitioning from the mild to the severe health states compared to the transition from not being ill to a mild illness health state. Respondents were willing to pay more to avoid a severe case over a mild case for one year; 114% more for HUS, 122% more for GBS, 138% more for IBS, and 166% more for ReA.

If unable to work most of the time without paid sick leave, the marginal WTP ranged from $6289 for IBS to $9872 for HUS to avoid a mild chronic illness; and to avoid a severe chronic illness the marginal WTP ranged from $8,394 for IBS to $11,352 for ReA.

The marginal WTP to avoid a chronic condition were not statistically significant when respondents were faced with being able to work some of the time and if sick leave was paid for the mild and severe health states. There is a large variation in the results of marginal WTP for ability to work/paid leave across all the illness models reflecting differences in the perceived severity of the conditions.

## Discussion

The study included a range of FBIs, which has allowed for an assessment of preferences for avoiding the health and loss of work disutility associated with different illnesses over short and long-terms using WTP. When using WTP to estimate the costs of these illnesses, the WTP will incorporate both the disutility of the illness to the individual and the impact of productivity losses (as measured by foregone wages). This means that WTP has a potential advantage compared with using market wages alone as it incorporates disutility to the individual as well as productivity losses. The inclusion of an attribute that varied ability to work, with and without paid sick leave, allowed us to consider how these factors affect individuals’ assessment of WTP. By considering paid and unpaid sick leave we are able to capture the extent to which this is a factor that would drive differences between productivity losses as measured by wages and productivity losses as measured by WTP. These estimated values could be used in economic evaluations that are based on a cost–benefit analysis framework.

The increases in the WTP for mild and severe health states were ordered as expected, with smaller incremental costs being associated with the transition from not being ill for any time to a mild illness health state, compared with transitions from the mild to the severe health states. The largest influence on the WTP to avoid a specified illness was observed when respondents were asked to consider their ability to work and the availability of paid leave. For all conditions, when sick leave was not available, participants expressed a stronger preference to avoid being ill and were willing to pay more to be able to work.

The MXL analyses revealed heterogeneity in preferences pertaining to cost, duration and ability to work without paid sick leave. Latent class modelling revealed distinct differences in the preferences observed across the classes with respect to duration of illness and costs of treatment. The preferences for the duration of illness were varied. For each LC models, preferences to not be ill for any duration of time were observed in two of the three classes; however, five of the models included a class where preferences for being ill for a duration of time were observed (class 2 mild and severe flu-like illness; class 1 mild ReA; class 3 mild HUS; class 1 of severe GBS). Preferences observed for most classes across the illnesses were for not paying for costs of treatment.

In the study by Hammitt and Haninger [41, 43] the WTP to reduce risk for a short-term morbidity was approximately $10,000 per statistical case avoided for adults and was twice as large for children. Our estimates for the marginal WTP to avoid a mild acute GI illness and flu-like illness were much smaller in magnitude. This may be explained by the fact that Hammitt and Haninger [43] used attributes based on risk reduction and mortality risk, whereas the attributes in this study focussed on the effect on work force participation and income, cost of treatment and duration of illness.

Respondents had divergent views on the consequences of the acute illnesses. Although we cannot compare the results of each illness directly, based on the MXL results, there is a preference to avoid the impacts of a mild GI illness in comparison to mild flu-like symptoms. The format of the DCE task presented two health states describing the acute illnesses that only differed on one descriptor (diarrhoea and fever), and this may indicate that there is a stronger preference to avoid an increase in frequency of experiencing diarrhoea in comparison to experiencing fever (as described in this study).

There are limitations of this work, as well as criticisms of DCE methods that should be acknowledged. Firstly, although DCEs have been shown to reasonably predict preferences, DCEs are fundamentally hypothetical and have been criticised for lacking external validity [14, 44, 45]. Our study was designed so that Treatment A was always more expensive and seen first, which was done to reduce cognitive burden for respondents. There is some evidence of left–right bias in previous DCEs [46]. In this study, the majority of respondents demonstrated trading behaviour between the two treatment profiles, but there was a small proportion of respondents who always chose the lowest cost (11%) or the shortest duration (4%). Furthermore, there was no opt-out option included. This decision was made during the design of the study, as we opted for forced choice to maximise the information gained from the choice sets. An opt-out could have been added for the acute illnesses, but for consistency across the illnesses we retained the forced choice framework. It is possible that the forced choice leads to higher WTP values. Other limitations in this study pertain to the WTP. Income disparities are a known issue, as the WTP measure is influenced by factors such as age, education, income, and ability to pay [47, 48]. Although we applied quotas by age, sex and location, we did not apply quotas based on income. True estimates of the WTP is conditional on taking up treatment. It is also important to note that the range of WTP estimates are determined in part by the range of costs included in the study, and so it may not reflect the maximum that a consumer is willing to pay for respondents whose WTP is outside of the costs for the average range in a market [49].

The findings need to be considered in the context of the health system, which may have affected preferences in relation to health care costs. If the illness was severe, it may be perceived that healthcare costs could be covered by other means, such as Medicare (Australia public subsidised health care) or via private health insurance. This may differ in other health systems; therefore there is a strong case for repeating this DCE internationally in different health settings. Further qualitative work investigating the reasons behind the different choices made across the conditions, different health profiles, and in the context of different healthcare systems may be beneficial. For example, some of the free text comments entered by respondents indicate that they were also considering other costs that may not be associated with treatment, or only focussed on the productivity costs, or the direct costs, due to out of pocket costs being high for them individually.

Lastly, it is important to note that our descriptions of illnesses were informed by the literature and a medical expert rather than the lived experience of patients. For rare conditions (eg GBS, ReA and HUS), this information would be a valuable addition, for example, by basing the descriptions on qualitative research such as interviews with patients to ensure relevant aspects are captured and the language is appropriate from a patient point of view.

There are notable advantages in using DCE techniques, such as enabling an efficient way of establishing preferences in the absence of revealed preference data and allowing for the relative importance of each attribute to be estimated individually [50]. Another strength of the study was the use of a large representative (in terms of age, gender and region) sample. Feedback from respondents regarding the ease of completion of the survey were comparable to other DCES valuing health states [46]. There is potential for WTP of various populations to be explored, and further work is required to explore the interpretation of these results to inform decision making.

The results suggest that the estimated WTP to avoid FBI is related to the amount of income lost when ill. Entitlements such as paid sick leave reduces the WTP estimates, suggesting that respondents are considering paid sick leave entitlements when they respond. When assessing the welfare changes associated with FBI, care should be taken to avoid double counting. The WTP with compensation reflects the impact of ill health, while the WTP without compensation reflects to some extent the individual income loss. The later would result in double counting if the productivity losses faced by firms were also included, therefore it may be more appropriate to estimate productivity losses separately.

## Conclusion and policy implications

Reliable estimates of the economic costs of specific foodborne infections are needed for policy makers to develop, prioritise and implement control measures with a net benefit to society. Most FBI can be prevented which reduces health care use and treatment costs. Preventative strategies are usually employed in food safety policy to reduce the incidence of FBI.

Lost productivity is one of the largest costs associated with the burden of FBI, but is often criticised for overestimating the actual burden of illness. Using WTP offers an alternative method for estimating costs, but it is important that this considers the effects of employment conditions, which influences values differently for short-term and long-term illnesses.

The findings from this DCE study illustrate that respondents value the consequences of the FBI based on important factors of severity of the illness and do consider the effect on productivity to be important in the long term. There are differences in preferences that translate into substantial differences in WTP to avoid an illness. These results have implications for estimating the burden and cost of FBI and suggest that as income loss is tempered by availability of paid sick leave.

## Figures and Tables

**Fig. 5:**
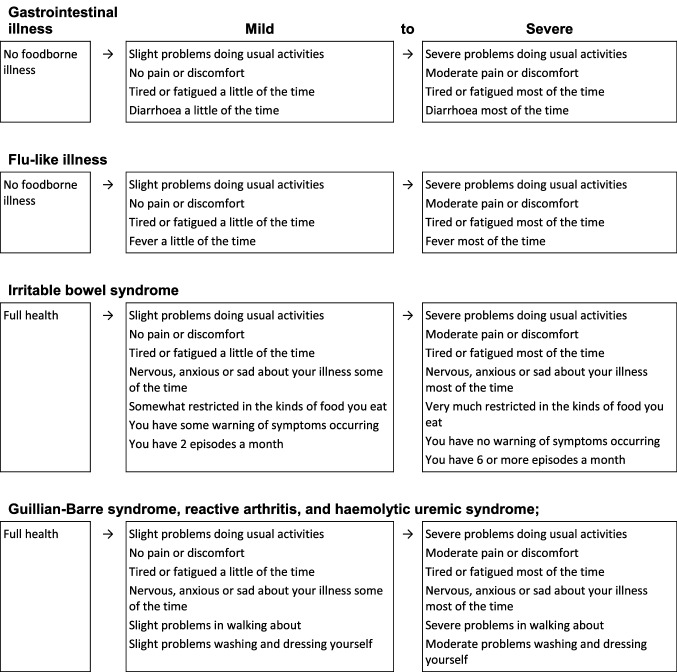
Health state descriptions

**Fig. 6:**
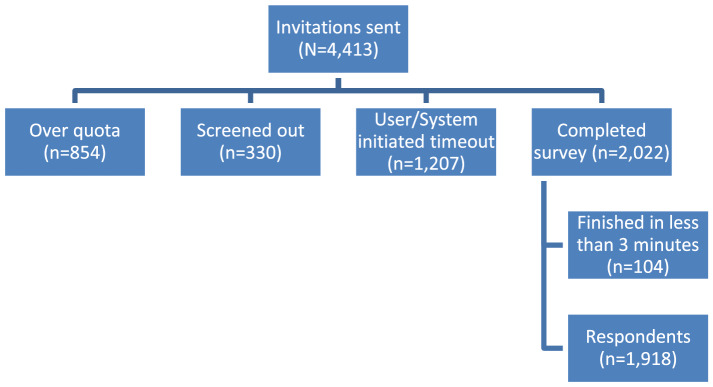
Disposition of respondents

## Data Availability

The datasets generated during and analysed during the current study are available from the corresponding author on reasonable request.

## Appendix

See Figs. 4, 5, 6 and Tables 4, 5, 6, 7, 8, 9, 10, 11

**Table 4:** Description of the illness

Condition	General description
Gastrointestinal illness	Imagine you have the symptoms of gastrointestinal illness which may include diarrhoea, fever, vomiting and nausea. You learn from your doctor that your symptoms are related to food you have recently eaten
Flu-like symptoms	Imagine you have symptoms which may include fever, muscle or body aches, headaches and are feeling tired. You learn from your doctor that your symptoms are related to food you have recently eaten
Irritable bowel syndrome	Irritable bowel syndrome (IBS) is a condition that results in pain and altered bowel habits, such as diarrhoea or constipation. People with IBS generally have abdominal cramping, lower belly pain, discomfort and bloating. The pain associated with IBS may be relieved by going to the toilet. The condition can last a long time and can affect life on a daily basis. For most people, the symptoms are intermittent. Symptoms associated with an episode of IBS generally last for two or so days
	What is an episode? An episode is diarrhoea or constipation that may last for a few days at a time
	For some people, the onset of an episode can be related to the types of food they eat, and there may be some warning of an episode, however, others may receive no warning of an episode. This may affect how or when they go out with friends or family, what they eat, or how they plan their day. This can result in social isolation
	When people with IBS go out, they often need to consider availability of toilet facilities. There are periods of time where sufferers have more frequent episodes than other times
	Irritable bowel syndrome doesn’t cause lasting damage and doesn’t contribute to the development of serious bowel conditions, such as cancer or colitis. There is no known cure for IBS and treatments are generally for management of individual symptoms
Guillian-Barre syndrome	Guillain-Barré syndrome is a rare and serious condition of the nervous system. It occurs when the body's immune system attacks part of the nervous system
	The symptoms of Guillain-Barré syndrome usually develop two to four weeks after a minor infection, such as a cold, sore throat or gastroenteritis (an infection of the stomach and bowel). Symptoms often start in your feet and hands before spreading to your arms and then your legs
	Initially, you may have: pain, tingling and numbness, progressive muscle weakness, co-ordination problems and unsteadiness (you may be unable to walk unaided)
	This can result in hospitalization or an emergency department visit
Reactive arthritis	Reactive arthritis is an illness which describes a group of symptoms including arthritis (swelling and pain of the joints), conjunctivitis (irritation and inflammation of the eye), and urethritis (urinary tract inflammation). In many patients, one or two of these symptoms may be present. Swelling and pain in the joints most commonly occurs in the knees and ankles, which may cause pain when walking or exercising
	The symptoms of reactive arthritis usually develop one to four weeks after a minor infection, such as gastroenteritis (an infection of the stomach and bowel)
	There is no known cure for reactive arthritis and treatments are generally for management of individual symptoms used to reduce inflammation and simple pain relief
Haemolytic uremic syndrome	Haemolytic uraemic syndrome (HUS) is a rare condition caused by a bacterial infection that releases toxins into the body. The illness stops the kidney’s filtering system from working
	The symptoms of HUS usually start as abdominal pains and diarrhoea lasting about a week. Fatigue and weakness then develop about two to eight days after the initial infection
	Appropriate treatment can lead to recovery, however, permanent kidney damage is possible, and in some cases HUS is fatal. Treatments may admission to hospital and in more severe cases admission to the intensive care unit. If chronic kidney disease develops, close observation in the hospital is required, and long-term treatments including dialysis, a kidney transplant and blood transfusions may be required

**Table 5:** Preference, acute illnesses (mixed logit models)

	Gastrointestinal illness (*N* = 670)				Flu-like illness (*N* = 662)			
	Mild		Severe		Mild		Severe	
	*β*	SD	*β*	SD	*β*	SD	*β*	SD
Cost of treatment^a^ (base: $0)	−7.413***		−8.193***		−7.351***		−8.436***	
Duration of illness (base: 1 day)	−0.087***	0.324***	−0.261***	0.599***	−0.056**	0.371***	−0.196***	0.579***
Ability to work and availability of paid sick leave (base: able to work)								
Unable to work WITH paid sick leave	−0.115	−0.531**	0.240**	−0.861***	−0.02	0.105	0.183	−1.001***
Unable to work WITHOUT paid sick leave	−1.086***	1.131***	−1.182***	1.470***	−1.147***	1.181***	−1.348***	1.412***
Obs	6700		6700		6620		6620	
Loglikelihood	−1938		−1824		−1863		−1789	
AIC	3889		3661		3741		3591	
BIC	3937		3709		3788		3639	

*SD* standard deviation

^a^Cost was scaled by *x*/1000

^*^*p* < 0.05; ***p* < 0.01; ****p* < 0.001

**Table 6:** Preferences, chronic illnesses (mixed logit models)

	Irritable bowel syndrome (*N* = 539)				Guillain Barre syndrome (*N* = 603)				Reactive arthritis (*N* = 607)				Haemolytic uraemic syndrome (*N* = 597)			
	Mild		Severe		Mild		Severe		Mild		Severe		Mild		Severe	
	*β*	SD	*β*	SD	*β*	SD	*β*	SD	*β*	SD	*β*	SD	*β*	SD	*β*	SD
Cost of treatment^a^ (base: $0)	−0.218***		−0.190***		0.159***		−0.143***		0.141***		−0.133***		−0.140***		−0.122***	
Duration of illness (base: 1 year)	−0.126**	0.594***	−0.259***	0.856***	−0.133***	0.653***	−0.264***	0.805***	−0.075**	0.546***	−0.187***	0.729***	−0.143***	0.579***	−0.268***	0.762***
Ability to work and availability of paid sick leave (base: able to work)																
Unable to work some of the time with paid sick leave	0.065	0.118	−0.277	0.431	0.051	0.730*	0.075	0.087	0.004	−0.468	−0.072	−0.319	−0.063	0.216	−0.032	−0.880**
Unable to work most of the time with paid sick leave	−0.004	0.037	−0.461*	−0.843**	−0.384**	−0.573	−0.274*	−0.734**	−0.282*	−0.304	−0.368**	0.087	−0.284*	−0.619*	−0.112	−0.281
Unable to work some of the time without paid sick leave	−0.751***	−1.149***	−1.583***	1.201***	−0.851***	−0.710**	−0.990***	−0.564	−0.836***	−0.772**	−0.981***	0.022	−0.987***	−0.454	−0.698***	−0.840**
Unable to work most of the time without paid sick leave	−1.374***	1.211***	−1.592***	1.599***	−1.490***	1.211***	−1.346***	−0.806*	−1.233***	−0.727*	−1.507***	−0.555	−1.378***	−0.981***	−1.386***	−0.684*
Obs	5390		5390		6030		6030		6070		6070		5970		5970	
Loglikelihood	−1386		−1381		−1609		−1562		−1647		−1570		−1619		−1577	
AIC	2794		2784		3240		3145		3316		3162		3260		3176	
BIC	2866		2856		3314		3219		3390		3236		3334		3250	

*SD* standard deviation

^a^Cost was scaled by *x*/1000

^*^*p* < 0.05; ***p* < 0.01; ****p* < 0.001

**Table 7:** Preferences, acute illnesses (latent class model)

	Gastrointestinal illness (*N* = 670)						Flu-like illness (*N* = 662)					
	Mild			Severe			Mild			Severe		
	Class 1	Class 2	Class 3	Class 1	Class 2	Class 3	Class 1	Class 2	Class 3	Class 1	Class 2	Class 3
Attributes												
Duration of illness (base: 1 day)	−0.131***	0.045	−0.334***	−0.449***	0.063	−0.103*	−0.222***	0.179***	−0.089	−0.03	0.153***	−0.319***
Cost of treatment (base: $0)	0.282	−15.312***	−9.510***	−2.382**	−15.868***	−7.321***	−9.545***	−12.443***	6.139	−10.349***	−12.808***	−3.632***
Ability to work and paid sick leave (base: able to work)												
Unable to work with paid sick leave	0.122	0.07	−0.629*	0.369**	0.439*	0.032	−0.049	0.127	0.04	−0.502	0.317	0.175
Unable to work with no paid sick leave	0.044	−0.714***	−2.435***	−0.099	0.179	−2.159***	−1.408***	−0.506	−0.261	−5.404	−0.214	−0.675***
Class membership parameters												
Gender	−0.598	0.031	0	−0.327	−0.141	0	0.930*	0.455	0	0.066	−0.035	0
Age	−2.131***	−0.966**	0	−0.192	−0.285	0	0.856*	0.778*	0	0.034	0.127	0
Income	0.291	−0.038	0	0.28	0.582	0	0.543	0.186	0	−0.215	−0.298	0
Employment status	0.054	−0.393	0	0.065	−0.586	0	−0.317	−0.958*	0	0.261	−0.544	0
Sick leave	−0.83	−0.391	0	−0.065	−0.007	0	−0.049	−0.086	0	0.201	0.473	0
Constant	1.419*	1.232*	0	0.352	0.232	0	0.181	0.589	0	−1.423**	−0.383	0
Class share	0.274	0.410	0.316	0.387	0.288	0.325	0.479	0.342	0.178	0.162	0.309	0.529
Unconditional probability	0.470	0.604	0.625	0.543	0.529	0.612	0.606	0.607	0.437	0.645	0.546	0.551
Conditional probability	0.565	0.771	0.748	0.746	0.788	0.754	0.687	0.827	0.647	0.902	0.823	0.662
Model statistics												
Mean posterior probability	0.797			0.834			0.820			0.867		
Loglikelihood	−1909			−1813			−1839			−1799		
CAIC	3998			3806			3859			3778		
BIC	3974			3782			3835			3754		
Observations	6700			6700			6620			6620		

Class membership parameters: gender: male = 0, female = 1; age: < 45 y = 0, ≥ 45 y = 1; income: < $52 k pa = 0,  ≥ $52 k pa = 1; employment status: unemployed = 0, employed = 1; sick leave: no sick leave = 0, paid sick leave = 1

Cost was scaled by *x*/1000

^*^*p* < 0.05; ***p* < 0.01; ****p* < 0.001

**Table 8:** Preferences, chronic mild illnesses (latent class models)

	Irritable bowel syndrome (*N* = 539)			Guillain Barre syndrome (*N* = 603)			Reactive arthritis (*N* = 607)			Haemolytic uraemic syndrome (*N* = 597)		
	Class 1	Class 2	Class 3	Class 1	Class 2	Class 3	Class 1	Class 2	Class 3	Class 1	Class 2	Class 3
Attributes												
Duration of illness (base: 1 year)	−1.148**	−0.170***	0.147	0.168	−0.160**	−0.281***	0.176*	−0.873***	−0.158***	−0.342***	−0.254***	0.193***
Cost of treatment (base: $0)	−0.049	−0.142***	−0.474***	−0.473***	−0.419***	−0.049***	−0.502**	−0.042	−0.111***	−0.03	−0.263***	−0.203***
Ability to work and paid sick leave (base: able to work)												
Unable to work some of the time with paid sick leave	−1.577	0.125	0.497	−0.712	0.547	−0.189	2.086**	0.942	−0.375**	−0.214	−0.586	0.431
Unable to work most of the time with paid sick leave	−1.463	−0.22	0.946	0.674	−1.263*	−0.446***	0.922	−0.149	−0.428**	−0.439*	−0.199	−0.184
Unable to work some of the time without paid sick leave	−2.798	−0.817***	−0.351	−0.398	−3.654**	−0.587***	−0.431	0.271	−1.085***	−0.463*	−2.454***	−0.204
Unable to work most of the time without paid sick leave	−2.945	−1.124***	−1.211*	−0.26	−4.312***	−0.885***	−1.202**	−0.399	−1.167***	−0.669***	−2.692***	−0.254
Class membership parameters												
Gender	−0.422	−0.073	0	−0.029	0.814*	0	−0.061	−0.496	0	0.397	0.545	0
Age	−1.424***	−0.744**	0	0.564*	0.961**	0	0.579*	−1.076**	0	−0.882***	0.236	0
Income	0.771*	0.267	0	−0.369	0.027	0	−0.014	0.918*	0	−0.083	0.565	0
Employment status	0.209	0.068	0	0.187	0.011	0	−0.509	−0.896	0	0.419	0.364	0
Sick leave	0.185	0.095	0	−0.304	−0.207	0	−0.074	−0.22	0	−0.266	−0.577	0
Constant	−0.665	0.732*	0	−0.665*	−1.852***	0	−0.391	−0.412	0	0.222	−0.674	0
Class share	0.116	0.529	0.355	0.300	0.208	0.492	0.353	0.134	0.513	0.361	0.311	0.328
Unconditional probability	0.433	0.599	0.659	0.598	0.649	0.511	0.638	0.456	0.563	0.500	0.626	0.581
Conditional probability	0.872	0.639	0.920	0.933	0.838	0.628	0.906	0.885	0.596	0.690	0.773	0.842
Model statistics												
Mean posterior probability	0.888			0.879			0.880			0.843		
Loglikelihood	−1355			−1562			−1595			−1585		
CAIC	2929			3347			3412			3393		
BIC	2899			3317			3382			3363		
Observations	5390			6030			6070			5970		

Class membership parameters: gender: male = 0, female = 1; age: < 45 y = 0, ≥ 45 y = 1; income: < $52 k pa = 0, ≥ $52 k pa = 1; employment status: unemployed = 0, employed = 1; sick leave: no sick leave = 0, paid sick leave = 1

Cost was scaled by *x*/1000

^*^*p* < 0.05; ***p* < 0.01; ****p* < 0.001

**Table 9:** Preferences, chronic severe illnesses (latent class models)

	Irritable bowel syndrome (*N* = 539)			Guillain Barre syndrome (*N* = 603)			Reactive arthritis (*N* = 607)			Haemolytic uraemic syndrome (*N* = 597)		
	Class 1	Class 2	Class 3	Class 1	Class 2	Class 3	Class 1	Class 2	Class 3	Class 1	Class 2	Class 3
Attributes												
Duration of illness (base: 1 year)	−0.511*	0.268	−0.150***	1.762**	−0.771***	−0.196***	0.208	−0.658***	−0.192***	−0.155***	−1.150**	−0.004
Cost of treatment (base: $0)	0.056	−1.484***	−0.114***	0.12	−0.027	−0.165***	−0.479*	0.011	−0.142***	−0.093***	−0.044	−0.686***
Ability to work and paid sick leave (base: able to work)												
Unable to work some of the time with paid sick leave	0.222	−4.441*	−0.173	−2.603*	0.388	−0.290*	−0.185	0.558	−0.513***	−0.255	−0.329	−0.184
Unable to work most of the time with paid sick leave	0.103	−9.523**	−0.422*	−0.702	−0.39	−0.457**	1.055	−0.512	−0.561***	−0.177	−0.497	1.115
Unable to work some of the time without paid sick leave	−0.383	−4.140**	−1.355***	0.233	−0.639	−1.204***	0.403	−0.371	−1.294***	−0.735***	−0.446	−0.874
Unable to work most of the time without paid sick leave	−0.603	−6.214**	−1.327***	−1.243	−0.891**	−1.379***	−0.518	−0.847*	−1.683***	−1.260***	−1.988*	−0.812
Class membership												
Gender	−0.314	0.013	0	0.031	−0.022	0	−0.446	−0.359	0	0.029	0.569*	0
Age	−0.639*	0.644*	0	−0.193	−0.353	0	0.701**	−0.524*	0	−0.897***	−0.817**	0
Income	0.041	−0.233	0	−0.238	0.205	0	−0.389	0.381	0	0.014	0.17	0
Employment status	0.16	0.098	0	−0.47	−0.275	0	−0.408	−0.451	0	0.161	0.504	0
Sick leave	−0.449	−0.534	0	0.358	0.175	0	0.134	0.126	0	−0.147	0.009	0
Constant	−0.106	−0.653*	0	−0.386	−0.229	0	−0.377	−0.054	0	1.035***	−0.158	0
Class share	0.249	0.294	0.457	0.308	0.234	0.458	0.286	0.258	0.456	0.462	0.272	0.266
Unconditional probability	0.492	0.595	0.587	0.525	0.500	0.573	0.577	0.503	0.581	0.556	0.541	0.537
Conditional probability	0.845	0.951	0.647	0.894	0.928	0.640	0.934	0.875	0.644	0.590	0.917	0.920
Model statistics												
Mean posterior probability	0.886			0.894			0.890			0.892		
Loglikelihood	−1333			−1539			−1519			−1530		
CAIC	2885			3300			3261			3281		
BIC	2855			3270			3231			3251		
Observations	5390			6030			6070			5970		

Class membership parameters: gender: male = 0, female = 1; age: < 45 y = 0, ≥ 45 y = 1; income: < $52 k pa = 0,  ≥ $52 k pa = 1; employment status: unemployed = 0, employed = 1; sick leave: no sick leave = 0, paid sick leave = 1

Cost was scaled by *x*/1000

^*^*p* < 0.05; ***p* < 0.01; ****p* < 0.001

**Table 10:** Acute illnesses: mWTP (95% CI) ($AUD 2017)

	Gastrointestinal illness (*N* = 670)		Flu−like illness (*N* = 662)	
	Mild	Severe	Mild	Severe
Duration of illness (base: 1 day)	$12 ($7, $16)	$32 ($25, $39)	$8 ($3, $13)	$23 ($17, $30)
Ability to work and paid sick leave (base: able to work)				
Unable to work with paid sick leave	$16 (−$5, $36)	−$29 (−$52, −$7)	$3 (−$17, $22)	−$22 (−$45, $1)
Unable to work without paid sick leave	$146 ($116, $177)	$144 ($112, $177)	$156 ($124, $188)	$160 ($126, $193)

**Table 11:** Chronic illnesses: mWTP (95% CI) ($AUD 2017)

	Irritable bowel syndrome (*N* = 539)		Guillain Barre syndrome (*N* = 603)		Reactive arthritis (*N* = 607)		Haemolytic uraemic syndrome (*N* = 597)	
	Mild	Severe	Mild	Severe	Mild	Severe	Mild	Severe
Duration of illness (base: 1 year)	$575 ($272, $877)	$1367 ($938, $1795)	$835 ($453, $1217)	$1852 ($1337, $2367)	$531 ($156, $907)	$1412 ($915, $1909)	$1025 ($630, $1419)	$2195 ($1596, $2794)
Ability to work and paid sick leave (base: able to work)								
Unable to work some of the time with paid sick leave	−$296 (−$1501, $909)	$1461 (−$91, $3013)	−$319 (−$2061, $1423)	−$529 (−$2409, $1351)	−$28 (−$1858, $1801)	$544 (−$1506, $2594)	$449 (−$1418, $2316)	$266 (−$2036, $2567)
Unable to work most of the time with paid sick leave	$18 (−$1518, $1554)	$2429 ($367, $4491)	$2409 ($764, $4054)	$1925 ($90, $3759)	$2001 ($347, $3654)	$2773 ($968, $4578)	$2032 ($262, $3801)	$915 (−$1048, $2878)
Unable to work some of the time without paid sick leave	$3437 ($1599, $5276)	$8347 ($5394, $11,301)	$5342 ($3660, $7024)	$6945 ($4913, $8977)	$5925 ($4121, $7729)	$7393 ($5416, $9371)	$7072 ($5192, $8953)	$5712 ($3392, $8032)
Unable to work most of the time without paid sick leave	$6289 ($4862, $7716)	$8394 ($6189, $10,599)	$9355 ($7202, $11,508)	$9442 ($7097, $11,787)	$8743 ($6697, $10,789)	$11,352 ($8770, $13,934)	$9872 ($7450, $12,294)	$11,338 ($8301, $14,375)
